# Valuable insights from the epacadostat plus pembrolizumab clinical trials in solid cancers

**DOI:** 10.1186/s12885-024-12432-1

**Published:** 2024-07-25

**Authors:** Tara C. Mitchell

**Affiliations:** Abramson Cancer Center, University of Pennsylvania Perelman School of Medicine, Philadelphia, PA USA

**Keywords:** Epacadostat, IDO-1 inhibitor, PD-1 inhibitor, Clinical trial

Checkpoint blockade has significantly improved outcomes and survival in patients with advanced cancer, with recent advances also seen in adjuvant and neoadjuvant therapy. The successful development of combination immunotherapy requires that a novel agent clearly improves outcomes over standard of care treatment. Several obstacles arise in the drug development process, however, and many valuable lessons become available from clinical trials, regardless of outcomes. In particular, despite over a decade passing since the initiation of the first-in-human clinical trials for PD-1 blockade, we still do not have ideal predictive markers of response and resistance for these agents. This absence of predictive markers has posed a challenge for combination therapy drug development; we are still not able to enrich trials for patients who are less likely to respond to PD-1 blockade alone, a great unmet need, which may have been insightful in the development of combination therapy with epacadostat, as well as other agents.

In this supplemental issue, the authors present final results of several clinical trials investigating the indoleamine 2,3 dioxygenase 1 (IDO1) inhibitor epacadostat versus placebo in combination with pembrolizumab in advanced solid tumors. In the case of IDO1 inhibition with epacadostat combined with PD-1 inhibition, while preliminary data from early phase I/II studies appeared promising [1–4], larger randomized trials did not confirm any benefit from the addition of epacadostat [5]. Several potential factors may have contributed to the lack of observed benefit with epacadostat in large, randomized trials.

The expected effect size of a novel combination must be high enough to clearly demonstrate significance of the combination over an agent known to be effective in a portion of the patient population. In the case of the studies included here, many of the patients enrolled on combination therapy trials may have benefitted from single agent therapy. For the first of these trials to be reported, which was in patients with advanced melanoma [5], it is likely that the efficacy of single agent pembrolizumab was underestimated based on historical controls for pembrolizumab response rates. At a 5-year follow up, the ORR among 655 advanced melanoma patients treated with pembrolizumab was 41%, while in the subset of patients who were treatment naïve, the response rate was 52% [6]. In contrast, the response rates previously reported from KEYNOTE-001 and KEYNOTE-006 were 38% and 33%, respectively [7, 8]. Randomized phase II studies help to give more accurate and objective efficacy data, rather than relying on historical controls in the decision to pursue larger randomized studies, especially since single arm phase I/II evidence can be misleading due to patient selection factors.

Furthermore, it is possible the epacadostat dose selected for randomized trials was inadequate. In the urothelial carcinoma study, for example, epacadostat 100 milligrams twice daily, when administered with pembrolizumab, did not normalize circulating kynurenine levels in most patients. The IDO1 enzyme catabolizes L-tryptophan to kynurenine, and plasma kynurenine was used as a biomarker for IDO1 inhibition. It may be that a sustained normalization of circulating kynurenine is a necessary result of effective IDO1 inhibition, but may still not be sufficient in effecting an improved anti-tumor immune response. Indeed, in a retrospective pooled analyses of several studies exploring different doses of epacadostat with PD-1 inhibitors, it was found that epacadostat doses < 600 mg bid, and higher than in the studies presented in this supplement, were still unable to maintain suppression of plasma kynurenine to normal levels [9]. The incorporation of pharmacokinetic and pharmacodynamic translational studies can help to solidify the case for further drug development when done in parallel with phase I/II studies. If on-target pharmacodynamic activity is consistently observed, it can be further developed as a potential biomarker assay during the drug development process, and ideally, incorporated prior to the initiation of larger registrational randomized phase III trials.

While IDO1 and PD-1 have non-overlapping mechanisms of action, it is rare that an agent with no demonstrated single agent activity results in synergistic anti-tumor efficacy when used in combination with an effective agent. Ideally, both agents would demonstrate single agent efficacy, as well as sustained pharmacodynamic on-target activity in a given tumor type prior to the development of combination therapy. Nonetheless, a purely synergistic contribution to efficacy is certainly possible. New emerging evidence suggests that IDO1 inhibition may be more complicated than previously thought. While epacadostat potently inhibits the catalytic activity of IDO1, it may enhance IDO1 intracellular signaling, promoting an immunosuppressive environment that may counteract, and work independently, of normalizing kynurenine levels [10, 11].

Currently, IDO is being investigated as a target in cellular/vaccine therapy approaches, including a PD-L1/IDO peptide vaccine in metastatic melanoma (NCT03047928), and another combination PD-L1 plus IDO targeted vaccine (NCT05155254) which includes an IDO peptide vaccine (IO102) which is currently being studied in several actively recruiting trials (clinicaltrials.gov) in melanoma, bladder, head and neck, and lung cancers. It remains to be seen if these alternate approaches to targeting IDO will lead to the realization of its potential role in promoting anti-tumor immunity.

In conclusion, we continue to learn valuable lessons from investigations into novel combination immunotherapies, and the epacadostat program offers many lessons. Before initiating future phase 3 programs, researchers may benefit from more extensive phase I and randomized phase 2 studies for dose selection, and to better understand the expected outcomes and appropriate biomarkers for the specific combinations. These considerations are integral to our collective goal for combination therapies to provide meaningful improvements in patient outcomes.

## Data Availability

Not applicable.

## Abbreviations

PD-1: Programmed death protein 1
PD-L1: Programmed death protein ligand 1
ORR: Objective response rate
